# Fatty Acid Metabolism in Ovarian Cancer: Therapeutic Implications

**DOI:** 10.3390/ijms23042170

**Published:** 2022-02-16

**Authors:** Hyunho Yoon, Sanghoon Lee

**Affiliations:** 1 Department of Medical and Biological Sciences, The Catholic University of Korea, Bucheon 14662, Korea; hyoon@catholic.ac.kr; 2 Department of Obstetrics and Gynecology, Korea University College of Medicine, Seoul 02841, Korea

**Keywords:** lipid metabolism, immune response, ovarian cancer, tumor microenvironment

## Abstract

Ovarian cancer is the most malignant gynecological tumor. Previous studies have reported that metabolic alterations resulting from deregulated lipid metabolism promote ovarian cancer aggressiveness. Lipid metabolism involves the oxidation of fatty acids, which leads to energy generation or new lipid metabolite synthesis. The upregulation of fatty acid synthesis and related signaling promote tumor cell proliferation and migration, and, consequently, lead to poor prognosis. Fatty acid-mediated lipid metabolism in the tumor microenvironment (TME) modulates tumor cell immunity by regulating immune cells, including T cells, B cells, macrophages, and natural killer cells, which play essential roles in ovarian cancer cell survival. Here, the types and sources of fatty acids and their interactions with the TME of ovarian cancer have been reviewed. Additionally, this review focuses on the role of fatty acid metabolism in tumor immunity and suggests that fatty acid and related lipid metabolic pathways are potential therapeutic targets for ovarian cancer.

## 1. Introduction

Fatty acids are important energy sources and structural components of cells in most species, including humans. Fatty acid oxidation, a lipid metabolic process, is essential for adenosine triphosphate (ATP) production and synthesis of new lipid metabolites [1]. Previous studies have reported that dysregulation of fatty acid metabolism is the etiological factor for various diseases, such as arteriosclerosis, diabetes, and fatty liver [2]. In particular, upregulated levels of fatty acids are associated with an increased risk of developing cancer because they regulate several biological functions, including maintaining the structure of cancer cell membranes and transducing oncogenic signals.

The fatty acid synthase (FASN or FAS) levels and the de novo synthesis of fatty acids are upregulated in several malignancies, such as breast [3], gastric [4], lung [5], liver [6], prostate [7,8], pancreatic [9], esophageal [10], and ovarian cancers [11], to maintain the uncontrolled growth and the increased survival rate of cancer cells. For example, free fatty acids promote estrogen receptor-alpha-positive breast cancer cell proliferation and aggressiveness through the activation of the mTOR pathway [12]. Previous studies have reported that upregulated FASN levels in several cancers are associated with increased fatty acid synthesis and poor prognosis [13]. FASN, a lipogenic enzyme, catalyzes the synthesis of new fatty acids using malonyl-CoA and acetyl-CoA as substrates [13]. Gouw et al. demonstrated that KRAS activated FASN promotes lung cancer cell proliferation [5]. One study evaluated 60, 20, and 10 squamous cell carcinoma, adenocarcinoma, and healthy esophageal tissue samples, respectively. The expression of FASN was detected in 90–95% of squamous cell carcinoma and adenocarcinoma samples, but not in healthy esophageal tissue samples. Additionally, FASN expression was positively correlated with esophageal cancer cell growth, migration, and tumorigenesis [10]. 

In cancer, fatty acid oxidation also contributes to oncogenic signal transduction, energy production, and cancer cell membrane architecture. MYC or JAK/STAT3-regulated fatty acid oxidation promotes triple-negative breast cancer (TNBC) cell growth, stemness, and chemoresistance, and provides novel therapeutic targets for TNBC [14,15,16]. D-bifunctional protein (DBP) is upregulated in prostate cancer and promotes fatty acid oxidation. Thus, DBP-mediated fatty acid oxidation is considered a potential oncogenic metabolic change that contributes to tumor progression [17]. Additionally, fatty acid oxidation promotes tumor chemoresistance and cell survival under hypoxic conditions. The inhibition of fatty acid oxidation increases the sensitivity of chemo-resistant cancer cells to mTORC1 inhibitors and paclitaxel in lung adenocarcinoma and chronic lymphocytic leukemia [18,19,20]. These studies suggested that fatty acid oxidation is a promising target for overcoming drug resistance in cancer treatment.

Recent studies on the interaction between fatty acids and tumor microenvironment (TME) have revealed that fatty acids play an important role in cancer cell survival by influencing tumor immunity. Fatty acid metabolism-stimulated tumor-infiltrating T lymphocytes enhanced the efficacy of melanoma immunotherapy [21]. Additionally, recent studies have demonstrated that regulatory T cells within tumors exhibit upregulated lipid contents, which resulted from the upregulation of glycolysis to support fatty acid synthesis and cell expansion [22]. These findings suggest that fatty acids alter tumor immunity by modulating the TME, which may be a promising therapeutic target in cancer immunotherapy. This review focuses on the role of fatty acid metabolism and its interaction with the TME of ovarian cancer. The therapeutic potential of fatty acid-associated lipid metabolism for ovarian cancer has also been discussed.

## 2. Fatty Acids

### 2.1. Source of Fatty Acids

Fatty acid metabolism is markedly altered in rapidly proliferating tumor cells, which results in increased ATP production [23,24]. Although most healthy cells prefer an exogenous source of fatty acid, tumor cells synthesize new fatty acids. Fatty acids are a major structural component of biological membranes and a component of complex lipids, such as triacylglycerols, membrane phospholipids, and signaling intermediates, including diacylglycerol, phosphoinositols, sphingosine, and phosphatidic acid [25]. These complex lipids are derived from acetyl-CoA, a building block for the de novo synthesis of fatty acids [1]. In the mitochondria, acetyl-CoA is generated from various nutrients, including sugars, proteins, and dietary fats.

### 2.2. FASN

Functional mammalian FASNs comprise homodimers with a conventional ‘head-to-tail’ structure. Each protein subunit comprises three catalytic domains at the N-terminus (ketoacyl synthetase [KS], malonyl acetyltransferase [MAT], and dehydratase) and four core domains at the C-terminus (enoyl reductase, ketoacyl reductase, acyl carrier protein [ACP], and thioesterase [TE]) [26,27]. The active arrangement of two identical proteins was mainly deduced from experiments with 1,3-dibromo-2-propanone that can crosslink the KS active site (Cys161 thiol) in one monomer with ACP (4′-phosphopantetheine thiol) in the other monomer [28,29]. Additionally, analysis of the catalytic activity of heterodimeric FASN, in which one subunit harbors mutations in all seven functional domains and the structural proximity between KS and MAT within the subunits of FASNs, revealed the formation of two coils in an overlapping arrangement [30,31].

In addition to the typical functions, human FASN plays important roles in the de novo biosynthesis of long-chain fatty acids, such as acetyl-CoA, malonyl-CoA, and 16 carbon (C16) palmitate from NADPH; the TE domain of FASN; and chain length-specific fatty acids, which leads to the release of palmitate through hydrolysis of the acyl-S-phosphopantetheine thioester [32]. The structure of the FASN TE domain is well conserved across species. Cryo-electron microscopy analysis has revealed the high specificity of TE for C16 to C18 fatty acyl substrates [33]. Human TE2, a type II thioesterase, regulates an FASN that promotes the premature release of short fatty acids during hydrolysis. Structural analysis revealed that human TE2 selectively interacts with the ACP domain of FASN, followed by interaction with a 4′-phosphopantetheinyl moiety attached to the ACP structure [34].

### 2.3. Fatty Acid-Binding Proteins (FABPs)

Studies examining the effect of fatty acids on cells have demonstrated that fatty acids regulate gene expression, growth and survival pathways, and linked signaling pathways for the metabolism of nutrient resources and mediate inflammatory responses [35,36,37]. FABP, a conserved protein, plays a pivotal role in lipid transport and metabolic reactions in various tissues and organs [38]. The FABP family comprises at least nine homologous proteins with specific tissue distribution patterns. These proteins are named primarily according to the tissue in which they are expressed. FABP family members include liver-specific, intestine-specific, heart-specific, adipocyte-specific, epidermis-specific, ileum-specific, brain-specific, myelin sheath-specific, and testis-specific FABPs [39]. The main functions of the cytoplasmic FABPs are to maintain the dynamic processes of cellular lipid metabolism, including lipolysis and peroxisome proliferator-activated receptor-gamma (PPARγ)-mediated adipogenesis. The roles of adipose-specific FABP (A-FABP) and epidermal-specific FABP in the pathogenesis of obesity-related diseases have been previously reported [40]. The expression of A-FABP, which is the best-characterized isoform, is regulated during adipocyte differentiation. Additionally, fatty acids, PPARγ agonists, and insulin regulate the transcription of A-FABP [41,42]. Previous studies focused on the development of high-affinity and selective chemicals targeting A-FABP have demonstrated that A-FABP functions as adipokines in obesity-associated breast cancer and mutant tumor cells with high A-FABP expression levels [43,44]. This indicates that targeting A-FABP is a potential therapeutic strategy for metabolic diseases.

### 2.4. Fatty Acid Transport Proteins (FATPs)

FATP, a transmembrane transport protein, allows long-chain fatty acids into cells, which, in turn, enhances fatty acid uptake. CD36, a member of FATP, has an extracellular binding site for fatty acids, an intracellular acyl-CoA synthetase active site and an ATP binding domain [45]. Fatty acids transported by CD36 are converted to secondary metabolites such as ceramides, diacylglycols, and inositol phospholipid derivatives. These metabolites play important roles in many biological functions, such as insulin resistance and cholesterol synthesis [46,47]. In addition to its fatty acid transport function, CD36 functions as a receptor for long-chain fatty acids. In taste cells, CD36-mediated linoleic acid inhibits serotonin and dopamine secretion by modulating Src kinase [48]. CD36 has also been implicated in some metabolic diseases, such as diabetes, Alzheimer’s disease, and cancer. For example, blockade of CD36 prevents atherosclerosis progression in high-fat diet mice [49,50]. In breast cancer, CD36 is highly expressed, and some studies have found that CD36 plays an essential role in cancer progression, migration and metastasis by regulating cell cycle and ERK1/2 signaling [51]. This suggests that CD36 could be a potential therapeutic target for treating CD36-related diseases.

## 3. Alteration of Fatty Acid Metabolism in Ovarian Cancer

### 3.1. Fatty Acid Metabolism in Ovarian Cancer

Ovarian cancer is one of the most common and malignant cancers among women. Epithelial ovarian cancer, which is the most common type of ovarian cancer that is initiated at the fallopian tube epithelium [52], is generally diagnosed at an advanced stage and is the most common cause of gynecological-related death [53]. Germ cell and stromal cell tumors of ovarian cancer are rare types of non-epithelial tumors, accounting for approximately 5–10% of all ovarian cancers [54,55]. These rare tumors are diagnosed at an early stage in young women and are associated with an improved 5-year survival rate [56,57]. High-grade serous ovarian cancer (HGSOC) is the most common histological type of ovarian cancer [58,59,60]. RNA sequencing (RNA-seq) and microarray data analysis of patients with HGSOC from The Cancer Genome Atlas revealed the following four subtypes of HGSOC based on the gene expression profiles: mesenchymal, immune response, proliferation, and differentiation [60,61]. HGSOC is often characterized by germline mutations in genes, such as those encoding p53 and BRCA. The mutated genes are genetic risk factors for ovarian cancer development [62,63]. Advances in targeted therapies, combination therapies, and immunotherapeutic agents have not markedly contributed to decreasing the death rates of ovarian cancer.

**Figure 1:**
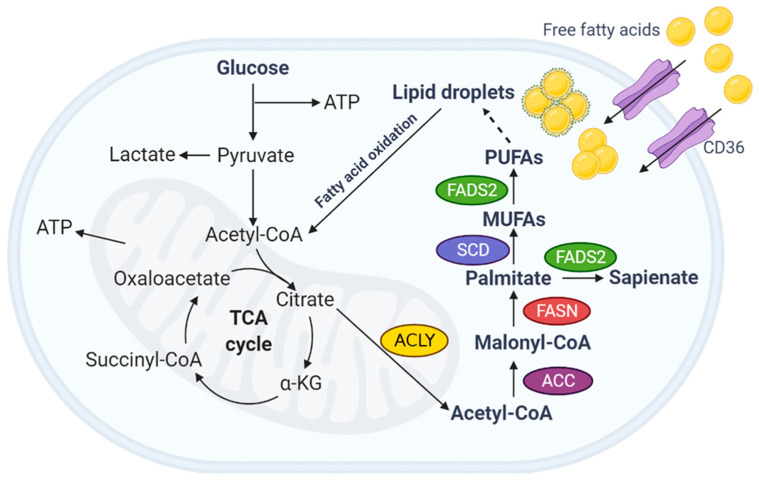
Fatty acid metabolism in the TME of ovarian cancer. Fatty acid-mediated lipid metabolism in TME is well controlled by cancer cells, adipocytes, and stromal cells with complex processes, leading to ovarian cancer metastasis and drug resistance. CAF, cancer-associated fibroblast; NK cell, natural killer cell; TAM, tumor-associated fibroblast; DC, dendritic cell; SREBP-1, sterol regulatory element binding protein 1; FABP, fatty acid binding protein; FASN, fatty acid synthase; SIK2, salt-inducible kinase 2; MCP-1, monocyte chemoattractant protein-1; TIMP-1, tissue inhibitor of metalloproteinase-1; and TGF-β1, transforming growth factor β1.

Accumulating studies indicate that plasma levels of fatty acid composition represent potential biomarkers for ovarian and other gynecological cancers [64,65]. Furthermore, recent studies have suggested that alterations in fatty acid metabolism may play a unique role in ovarian cancer pathogenesis and aggressiveness (Figure 1). FASN, which is upregulated in ovarian cancer tissues, is associated with poor prognosis and survival [66]. In ovarian clear cell carcinoma, cancer grade was significantly correlated with FASN expression [66]. Grunt et al. reported that cell membrane FASN-mediated phospholipids interact with receptor tyrosine kinases, including ErbB2 (HER2/neu), which are upregulated in ovarian cancer [67]. This interaction activates the phosphoinositide-3-kinase (PI3K)-mTOR pathway, which promotes the proliferation and survival of ovarian cancer cells [66,67,68]. The upregulated expression of FASN ovarian cancer cell lines and primary cultures increases de novo fatty acid synthesis, cell growth, and cell viability, and enhances chemoresistance to cisplatin [69,70]. These studies suggest that FASN is a metabolic marker for ovarian cancer development and progression.

Stearoyl-CoA desaturase-1 (SCD1) is an endoplasmic reticulum enzyme that catalyzes the synthesis of saturated fatty acids (e.g., oleates and palmitolates) from mono-unsaturated fatty acids (e.g., stearoyl-CoA and palmitoyl-CoA). Previous studies have demonstrated that SCD1 is a biochemical hallmark of cancer cells and that it modulates fatty acid composition in cancer [71]. The expression of SCD1 is upregulated in ovarian cancer stem cells [72]. Treatment with SCD1 inhibitors suppresses the growth of ovarian cancer stem cells in a mouse model. Mechanistic studies revealed that NF-kB can directly regulate the transcription of SCD1 [73]. Additionally, upregulated SCD1 expression levels protected ovarian cells against ferroptosis, an iron-mediated oxidative damage that inhibits the growth of ovarian cancer cells [74,75]. This suggested that targeting SCD1 is a potential therapeutic strategy for ovarian cancer.

Exogenous fatty acid metabolism induced by human adipocytes is also strongly associated with cancer progression and metastasis. Co-culture of human omental adipocytes with ovarian cancer cells promoted the growth, homing, migration, and invasion of ovarian cancer cells by providing fatty acids [76,77]. In omental metastases, FABP4, a type of lipid chaperones, was detected at the interface between adipocytes and tumor cells [78]. FABP4 modulates lipid metabolism of ovarian cancer cells by destroying tumor-infiltrating dendritic cells, thereby interfering with anti-tumor immunity, resulting in poor prognosis of ovarian cancer [76,78]. These studies suggested that FABP4 functions as a key mediator between adipocytes and cancer progression. Thus, FABP4 is a potential therapeutic target for ovarian cancer.

### 3.2. Fatty Acid Metabolism in the TME of Ovarian Cancer

Adipocytes in the TME serve as a major source of fatty acids. In the TME, adipocyte-derived lipids, including fatty acids, affect cancer cells and various peripheral cells, such as cancer-associated fibroblasts, dendritic cells, macrophages, and immune cells. Cancer cells stimulate adipocytes with inflammatory cytokines, which are closely related to lipid production. Yu et al. demonstrated that interleukin-17A (IL-17A), a pro-inflammatory cytokine, promoted the growth and metastasis of ovarian cancer by regulating fatty acid metabolism in adipocytes, especially regulating fatty acid uptake by cancer cells. Human recombinant IL-17A induces fatty acid uptake by upregulating FABP4 expression in OvCa cells and consequently contributes to the progression and metastasis of ovarian cancer cells [79]. Specifically, IL-17A activated STAT3 phosphorylation to promote FABP4 expression, thereby increasing ovarian cancer cell proliferation. Immune cell-derived cytokines, including IL-1β and transforming growth factor β1 (TGF-β1), are reported to promote the release of saturated fatty acids by stimulating lipolysis in adipocytes. These saturated fatty acids activate TLR4 signaling in macrophages, which leads to the stimulation of the production of pro-inflammatory mediators involved in conferring chemical resistance to the tumor cells [80,81].

Adipokines, such as IL-6, IL-8, monocyte chemoattractant protein-1 (MCP-1), tissue inhibitor of metalloproteinase-1 (TIMP-1), and adiponectin in the ovarian cancer microenvironment can promote cancer cell growth by activating fatty acid production in adipocytes [76]. In addition, adipokines were involved in ovarian follicle development and cancer by regulating PI3K/AKT, AMP-activated protein kinase (AMPK), and peroxisome proliferator-activated receptor (PPAR) signaling pathways [82]. Additionally, polyunsaturated fatty acids, such as linoleic acid accumulated in the ovarian cancer microenvironment, can activate peroxisome proliferator-activated receptor β/δ (PPARβ/δ) signaling in tumor-associated macrophages (TAMs) [83]. Activation of PPARβ/δ, which belongs to the nuclear receptor group, is one of the hallmarks of cancer [84]. Previous studies have reported that activated PPARβ/δ is a master regulator of adipocyte differentiation and that it modulates fatty acid storage and glucose metabolism [85]. In ovarian cancer, TAMs regulate metabolic function through PPARβ/δ and some signature genes (e.g., LRP5, CD300A, MAP3K8, and ANGPTL4) associated with immune regulation and tumor progression that correlate with short relapse-free survival in serous ovarian cancer [83].

Adipocyte-associated molecules are reported to regulate cancer metastasis by regulating cancer cell metabolism. Miranda et al. demonstrated that salt-inducible kinase 2 (SIK2) has an essential role in adipocyte-induced ovarian cancer metastasis [86,87]. SIK2, which is upregulated in adipocyte-rich metastatic deposits, regulates both phosphatidylinositol 3-kinase (PI3K) and acetyl-CoA carboxylase 1 (ACC1)-mediated fatty acid oxidation and consequently promotes omental metastasis [86]. These studies suggested that lipid metabolism in the TME is regulated by cancer cells, lipid cells, and surrounding cells through a complex process. Therefore, elucidation of the interactions between ovarian cancer cells and surrounding stromal cell types in the ovarian cancer microenvironment will provide useful insights for the development of novel therapeutic approaches for ovarian cancer.

### 3.3. Fatty Acid-Mediated Ovarian Cancer Immunity

Fatty acids secreted by tumor-associated stromal cells, including adipocytes, may exert tumor-promoting effects on various immune cells, such as macrophages, natural killer (NK) cells, dendritic cells, neutrophils, and T cells recruited to the TME. Reprogramming of lipid metabolism in tumor cells caused by these interactions may provide cells with a survival advantage during cancer progression and metastasis. For example, T cell activation or differentiation is closely related to fatty acid synthesis and oxidation via the mTOR-sterol regulatory element binding protein (SREBP) pathway [88]. Some recent studies have demonstrated that aberrant activation of FASN can impair the anti-tumor immunity in cancer immunotherapy [89]. Jiang et al. reported upregulated levels of unsaturated fatty acids, saturated fatty acids, and triacylglycerols in the ascites of ID8 (mouse ovarian surface epithelial cell line)-bearing mice exhibiting FASN overexpression. In the TME, aberrant lipid accumulation impairs tumor-infiltrating dendritic cells, which leads to the inhibition of anti-tumor T cell infiltration [89]. This suggested that aberrant overexpression of FASN is correlated with immunosuppressive status in ovarian cancer.

The infiltration and differentiation of TAMs are positively correlated with all stages of tumor progression, angiogenesis, and metastasis because they secrete various cytokines and chemokines and regulate the anti-tumor immune responses of T and NK cells [90,91]. Previous studies have revealed a correlation between obesity and ovarian cancer incidence, progression, and metastasis [92]. Liu et al. demonstrated that obesity promotes ovarian cancer metastasis by increasing lipogenesis and decreasing the infiltration of M1 macrophages that initiate an immune response against bacteria and viruses [93,94]. In obesity, the expression of SREBP-1, a transcription factor involved in fatty acid synthesis in ovarian cancer cells, is upregulated, which leads to increased accumulation of new fatty acids and enhanced fatty acid transport [93]. De novo fatty acid synthesis promoted ovarian metastatic potential by increasing vascularity and downregulated the infiltration of M1 macrophages. These findings are not consistent with the previously reported role of macrophages. However, the inverse correlation between M1:M2 ratio of TAMs and tumor stage was reported to be associated with poor overall survival [95]. Further studies are needed to investigate fatty acid and related macrophage-based immune responses in ovarian cancer.

## 4. Fatty Acid Metabolism-Targeted Therapeutic Strategies for Ovarian Cancer

### 4.1. FASN Inhibitors

Most cancers depend on the synthesis of new fatty acids. Hence, FASN is a potential therapeutic target for cancer. Previous studies have reported that the inhibition of FASN exerts growth-inhibitory effects on ovarian cancer [96,97]. Treatment with C75 and G28UCM, which are the synthetic inhibitors of FASN, decreased ovarian cancer cell growth, and induced apoptosis [98]. Mechanistic studies revealed that C75 markedly inhibited lipogenesis and downregulated the oncogenic PI3K-AKT signaling pathway [67,99]. Cerulenin, a specific FASN inhibitor, suppresses the expression of HER2/neu in cancer [100]. Treatment with cerulenin markedly inhibited fatty acid biosynthesis in a tumor xenograft model of ovarian cancer and increased the survival rates [101]. Additionally, treatment with cerulenin potentiated the anti-tumor immune responses of cytotoxic T cells and consequently inhibited tumor growth in the xenograft models of ovarian cancer [89]. Thus, cerulenin has potential applications in ovarian cancer immunotherapy. Orlistat, a potent pancreatic lipase inhibitor that was approved by the Food and Drug Administration in 2010 to treat obesity, prevents the absorption of fat from the diet in humans [102]. Previous studies have reported that orlistat exerts growth-inhibitory effects against various cancers by inhibiting FASN [13]. Papaevangelo et al. reported that orlistat inhibited fatty acid metabolism in ovarian cancer cells and that orlistat potentiated the growth-inhibitory effects of cisplatin against platinum-resistant ovarian cancer cells in vivo by inducing apoptosis and necrosis [70]. C93, a FASN inhibitor, inhibited the growth of carboplatin/paclitaxel-resistant ovarian cancer cells [103]. Treatment with C93 induced apoptosis and mitigated cisplatin resistance in ovarian cancer cells [69]. These results suggest that FASN is a potential therapeutic target for ovarian cancer.

### 4.2. Fatty Acid Uptake Inhibitors

Exogenous fatty acid intake can promote cancer progression and metastasis. Most of these processes are mediated by CD36, low-density lipoprotein receptor, and FABP in the cancer cell membrane, which are potential therapeutic targets for cancer. Preclinical studies have reported that treatment with anti-CD36 antibodies significantly exerted anti-tumor or anti-metastatic effects [104]. Treatment with anti-CD36 monoclonal antibodies decreased tumor burden in mouse xenografts of ovarian cancer [105]. Jayawardhana et al. engineered fatty acid-like Pt(IV) prodrugs (FALPs), which inhibit CD36-dependent fatty acid uptake [106]. FALPs exerted potent-growth-inhibitory effects against cisplatin-resistant ovarian cancer cells by promoting mitochondrial damage [106]. BMS309403, a small-molecule inhibitor of FABPs, including FABP4, competitively inhibits the binding of endogenous fatty acids by interacting with the fatty acid-binding pocket [107]. In ovarian cancer cells, BMS309403 significantly inhibited lipid accumulation and adipocyte-mediated omental metastasis [76]. Additionally, treatment with the FABP4 inhibitor suppressed ovarian cancer cell proliferation and omental colonization and increased the sensitivity of cancer cells to carboplatin [108]. This suggested that targeting the free fatty acid uptake pathway is a potential therapeutic strategy for ovarian cancer.

### 4.3. Other Inhibitors Targeting Fatty Acid Metabolism

A939572, a potent small-molecule inhibitor of SCD1, induces apoptosis and inhibits the proliferation of cancer cells, including kidney, bladder, liver, colon, and thyroid cancer cells [109,110,111,112]. SCD1, which protects the cancer cells against ferroptosis, is a potential therapeutic target for ovarian cancer. Treatment with A939572 significantly potentiated the growth-inhibitory effects of the ferroptosis inducers RSL3 and erastin on ovarian cancer cells and in vivo xenograft models [75].

The expression of SIK2 is upregulated in adipocyte-rich metastatic deposits in ovarian cancer and is strongly correlated with abdominal metastasis. Zhou et al. examined the effects of ARN-3236, a small-molecule inhibitor of SIK2, on ovarian cell growth in vitro and in vivo [86,113]. Treatment with ARN-3236 decreased ovarian cancer growth and enhanced the response to paclitaxel chemotherapy [113]. This suggested that targeting SIK2 is a potential therapeutic strategy for cancer.

Miranda et al. demonstrated that downregulated levels of AMPK promote peritoneal metastasis of ovarian cancer by activating carnitine palmitoyltransferase 1 (CPT1) through the regulation of acetyl-CoA carboxylase phosphorylation [86]. One study examined the efficacy of a metabolic inhibitor cocktail containing transforming growth factor beta-activated kinase 1 (TAK1) (AMPK activator) and FASN synthase inhibitors against ovarian cancer cells [97]. Treatment with the inhibitor cocktail decreased ovarian cancer metastasis and aggressiveness by inhibiting the mTOR and TAK1 signaling pathways [97]. This indicated that targeting AMPK-mediated lipid metabolism is a potential therapeutic strategy to mitigate peritoneal metastasis in ovarian cancer (Table 1).

## 5. Conclusions and Perspective

**Figure 2:**
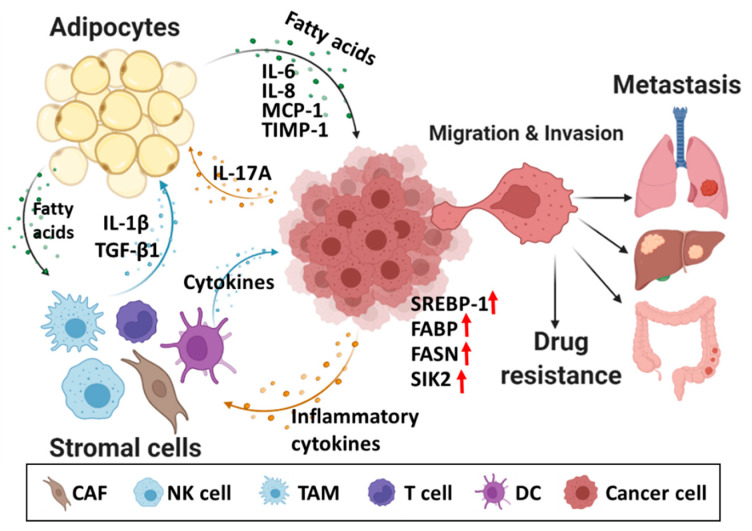
Fatty acid metabolism in the TME of ovarian cancer. Fatty acid-mediated lipid metabolism in TME is well controlled by cancer cells, adipocytes and stromal cells with complex processes, leading to ovarian cancer metastasis and drug resistance. CAF, cancer-associated fibroblast; NK cell, natural killer cell; TAM, tumor-associated fibroblast; DC, dendritic cell; SREBP-1, sterol regulatory element binding protein 1; FABP, fatty acid binding protein; FASN, fatty acid synthase; SIK2, salt-inducible kinase 2; MCP-1, monocyte chemoattractant protein-1; TIMP-1, tissue inhibitor of metalloproteinase-1; TGF-β1, transforming growth factor β1.

Fatty acid-induced lipid metabolic reprogramming is associated with increased incidence and aggressiveness of ovarian cancer. Several studies have elucidated the mechanisms underlying fatty acid-mediated ovarian cancer progression, recurrence, and metastasis. Adipocyte-derived fatty acids can alter tumor immunity by recruiting immune cells to the TME. This fatty acid-mediated lipid metabolic reprogramming provides survival advantages to tumor cells during therapy and metastasis (Figure 2). Fatty acid metabolism in ovarian cancer is a complex process that includes lipid absorption, lipid synthesis, and fatty acid oxidation. The roles of enzymes involved in fatty acid synthesis and lipid absorption in ovarian cancer pathogenesis and chemoresistance have been characterized. Thus, the modulation of these enzymes is a potential, novel therapeutic strategy for ovarian cancer. Several preclinical and clinical trials targeting these enzymes have demonstrated improved treatment outcomes and prevention of further spread and progression of cancer. However, some FASN inhibitors were associated with neuronal stem cell dysfunction and serious side effects, including decreased food intake and weight loss in mice [114,115]. These findings indicate that regulated crosstalk between ovarian cancer cells and surrounding cells in the TME can modulate lipid metabolic processes by reprogramming fatty acid metabolism and consequently promotes ovarian cancer proliferation, invasion, metastasis, and drug resistance. Although the clinical safety of fatty acid-targeted drugs is a concern, fatty acid and related lipid metabolic pathways are potential therapeutic targets for ovarian cancer.

## Figures and Tables

**Table 1:** Therapeutic strategies targeting fatty acid metabolism for ovarian cancer.

	Drug	Effects and Features	References
Fatty acid synthase inhibitors	C75	Abrogating lipogenesis; downregulating PI3K-AKT signaling pathway; antitumor effects	[67,98,99]
	G28UCM	Decreasing cell growth and inducing apoptosis	[98]
	Cerulenin	Also known as an inhibitor of HER2/neu; inhibiting fatty acid biosynthesis in a xenograft model; enhancing antitumor immunity of T cells; inhibiting tumor growth and increasing mice survival	[89,100,101]
	Orlistat	Potent inhibitor of pancreatic lipase; FDA-approved for anti-obesity; abolishing fatty acid metabolism; combination treatment with cisplatin enhanced in vivo efficacy	[13,70]
	C93	Inhibiting growth of carboplatin/paclitaxel-resistant ovarian cancer cells; re-sensitizing cisplatin resistant cancer cells; antitumor effects in ovarian cancer	[69,103]
Fatty acid uptake inhibitors	Anti-CD36 monoclonal antibody	Significant anti-tumor or anti-metastatic efficacy in preclinical studies; reduced tumor burden in mouse xenografts of ovarian cancer	[104,105]
	FALPs	Inhibiting CD36-dependant fatty acid uptake; increased mitochondrial damage by FALPs decreased cell growth in cisplatin-resistant ovarian cancer cells	[106]
	BMS309403	Small molecule inhibitor of fatty acid binding proteins; competitive inhibitors of the binding of endogenous fatty acids; reducing adipocyte-mediated omental metastasis; increasing the sensitivity of ovarian cancer cells to carboplatin	[76,107,108]
Other inhibitors targeting fatty acid metabolism	A939572	Potent small molecule inhibitor of SCD1; enhancing the anticancer effects of the feroptosis inducers, RSL3 and erastin, on ovarian cancer cells and in vivo xenograft models	[75]
	ARN-3236	Small molecule inhibitor of SIK2; Inhibiting ovarian cell growth in vitro and in vivo; showing improved response to paclitaxel chemotherapy	[86,113]
	TAK1	AMPK activator and fatty acid synthase inhibitor; reducing ovarian cancer metastasis by inhibiting mTOR and TAK1 signaling pathway	[86,97]

## Data Availability

Not applicable.
